# Optical Characteristics of a New Molecular Complex: “Nafion–Colloidal CdSe/CdS/ZnS Nanocrystals”

**DOI:** 10.3390/polym16142092

**Published:** 2024-07-22

**Authors:** Svetlana L. Timchenko, Sergey A. Ambrozevich, Evgenii N. Zadorozhnyi, Nikolai A. Zadorozhnyi, Alexander V. Skrabatun, Evgenii A. Sharandin

**Affiliations:** 1 Department of Fundamental Sciences, Bauman Moscow State Technical University, 2-nd Baumanskaya Street 5, 105005 Moscow, Russia; s.ambrozevich@mail.ru (S.A.A.); eugenezador@gmail.com (E.N.Z.); nikazador@mail.ru (N.A.Z.); alskrabatun@mail.ru (A.V.S.); shar@bmstu.ru (E.A.S.); 2 P.N. Lebedev Physical Institute of the Russian Academy of Sciences, Leninskiy Prospekt 53, 119991 Moscow, Russia

**Keywords:** colloidal nanocrystals, Nafion, luminescence, absorption spectroscopy, laser emission spectroscopy, toluene

## Abstract

Here, the optical properties of the Nafion polymer membrane containing colloidal CdSe/CdS/ZnS nanocrystals embedded by diffusion have been studied. The CdSe/CdS/ZnS nanocrystals have a core/shell/shell appearance. All experiments were carried out at room temperature (22 ± 2) °C. A toluene solution was used to provide mobility to the active sulfone groups of the Nafion membrane and to embed the nanocrystals inside the membrane. The diffusion process of colloidal CdSe/CdS/ZnS nanocrystals into Nafion proton exchange membrane has resulted in a new molecular complex “Nafion–colloidal CdSe/CdS/ZnS nanocrystals”. The kinetics of the nanocrystals embedding into the membrane matrix was investigated using luminescence analysis and absorption spectroscopy techniques. The embedding rate of CdSe/CdS/ZnS nanocrystals into the Nafion polymer membrane was approximately 4·10^−3^ min^−1^. The presence of new luminescence centers in the membrane was proved independently by laser emission spectroscopy. The luminescence spectrum of the resulting molecular complex contains intensity maxima at wavelengths of 538, 588, 643 and 700 nm. The additional luminescence maximum observed at the 643 nm wavelength was not recorded in the original membrane, solvent or in the spectrum of the semiconductor nanoparticles. The luminescence maximum of the colloidal CdSe/CdS/ZnS nanocrystals was registered at a wavelength of 634 nm. The intensity of the luminescence spectrum of the membrane with embedded nanocrystals was found to be higher than the intensity of the secondary emission peak of the initial nanocrystals, which is important for the practical use of the “Nafion–colloidal nanocrystals” complex in optical systems. The lines contained in the luminescence spectrum of the membrane, which has been in solution with colloidal nanocrystals for a long time, registered upon its drying, show the kinetics of the formation of the molecular complex “Nafion membrane–nanocrystals”. Colloidal nanocrystals located in the Nafion matrix represent an analog of a luminescent transducer.

## 1. Introduction

The development of methods for creating light energy is an are urgent practical problem. For example, the process of embedding nanoparticles in optically transparent media, in polymer films in particular, is used in the production of full-color displays [1,2,3]. This leads to the excitation of spectrally narrow luminescence lines. Determination of the degree of influence of the doping process on the physical characteristics of radiation transducers, particularly those based on polymer matrices, is practically important at this stage [4,5,6]. Semiconductor crystals implanted in a polymer matrix induce charge carrier transport. As a result, the influence of nanocrystals on spatial charge separation is possible in such systems [6]. Nanocrystals embedded in organic semiconductor matrices can act as centers of radiative recombination in the structures of organic light-emitting diodes [7].

The emission spectrum of light-emitting systems can be regulated by embedding nanocrystals of different sizes [7]. The embedded nanocrystals exhibit good photostability, a high photo-decolorization threshold and a high quantum efficiency. The type of passivating agent on the nanocrystal’s surface and the composition of the organic matrix in which these nanoobjects are embedded significantly affect their electrical and luminescent characteristics. For identical nanoparticles whose structure is core/shell/shell such as CdSe/CdS/ZnS, changing the type of passivator leads to significant differences in the dipole–dipole energy transfer rates.

The authors of [8] were able to record the main luminescence line and a two-phonon replica in their study of photoluminescence of CdSe nanocrystals in a glass matrix at a low temperature and using selective excitation. A practically important dependence of the exchange energy in nanocrystals on their size has been established. The value of the electron–hole exchange energy obtained from the energy separation between the excitation energy and the luminescence line reaches 24 meV in nanocrystals with a diameter of 30 Å. At the same time, radiative recombination is made possible by phonon virtual transition to a confined exciton state. Systems containing luminescent nanocrystals are used in electro-optical transducers; therefore, information on the quantum characteristics of this system is practically useful. In [9], the ionization potentials, electron affinity and quantum confinement in CdSe nanocrystals were determined by cyclic voltammetry.

By the end of the XX century, the technology for the synthesis of spherical nanocrystals was realized. The technology was based on the precipitation reaction in a homogeneous aqueous solution with the participation of surface-active and polymeric stabilizers (see the works of Brus L. and Henglein A. [10,11]). The desire to synthesize high-quality nanocrystals, to increase the photoluminescence quantum yield of the system and to reduce the contribution of defects led to an increase in the synthesis temperature [12]. The ZnS coating, with a thickness of (6 ± 3) Å, passivates and protects the surface, which is confirmed by enhanced and stable luminescence at room temperature with a quantum yield of 50%.

Initially, the synthesis of core/shell nanocrystals was carried out by growing the core itself, followed by a purification step. Then, the shell growth reaction was carried out. Using methods in which the intermediate purification stage is absent, it was possible to obtain core/shell nanoparticles from various combinations of materials—CdSe/ZnSe [13], CdSe/CdS [14] and InP/ZnS [15].

The controlled synthesis of high-quality CdS/ZnSe/ZnS core/shell1/shell2 nanocrystals was investigated in [16]. The regulation of the photoluminescence range was achieved by using CdS nanocrystals of different sizes and ZnS shell thicknesses as cores. Due to the growth of thick ZnS shells, the photoluminescence quantum yield of these nanocrystals was 50–60%. For CdS/ZnSe nanocrystals with type II optical characteristics, the secondary emission maximum ranged from 500 nm to 630 nm [16].

Obtaining selective light emission generated by a multilayer quantum system consisting of CdSe and CdS nanocrystals with different properties requires controlling the deformation of nanocrystals by adjusting the thickness of the potential well and barrier [17]. The strain compensation was performed by using an optimized ZnS outer shell, which increased the quantum yield of CdSe up to 48%. The authors of [17] proved that the composition of the luminescence spectrum can be modified by adding an inner ZnS barrier layer to block charge transfer and excitons between the QW and the CdS nucleus.

The authors of [18] proposed a concept of a hybrid nanofluidic device based on nanocrystals/III-nitrides. Direct electro-optical pumping of nanocrystals was carried out from electrically controlled InGaN/GaN nanofluidiodes, which were the primary sources of radiation. Electro-optical pumping of CdSe-nanocrystals was realized, which was carried out as a non-contact approach. The photon–photon conversion efficiency was 27%. In [19], the influence of GaN-based LED array geometry and the materials used in the contact lines on the final size and shape of the backlight spot was investigated. GaN-based light-emitting diodes were found to operate efficiently down to the nanoscale.

One of the important applications of semiconductor nanoparticles is in the diagnosis of various diseases. In this case, nanoparticles play the role of luminescent markers. The use of organic dyes for this purpose is limited as they can be toxic. In this respect, nanoparticles, when properly passivated, have a competitive advantage [20]. However, nanoparticles are quite large entities and are comparable to the pore size of cell membranes. Therefore, whether they can reach the target to be visualized fast enough and whether this will lead to the degradation of the properties of the nanoparticles themselves should be clarified. This question can be addressed in “in vivo” experiments. However, the answer to this question can be answered “in vitro” by using the Nafion matrix [21,22], which has already demonstrated its effectiveness as a model of biological tissues.

Nafion (C_7_HF_13_O_5_S C_2_F_4_) membrane is produced by the copolymerization of perfluorinated vinyl ether monomer with tetrafluoroethylene [23,24] and consists of perfluorinated vinyl ether groups terminated with tetrafluoroethylene (Teflon)-based sulfone groups. Teflon is a highly hydrophobic membrane matrix. The sulfone groups in the membrane are fairly hydrophilic compounds. The Nafion polymer membrane matrix is biocompatible and flexible. The membrane has good mechanical and chemical stability due to the inertness of its fluorocarbon backbone [24]. A large number of studies have been devoted to the study of the effect of impregnation of the Nafion membrane with water [25,26] and aqueous solutions, for example, methylene blue [27,28], which is accompanied by the manifestation of the effects of forward and reverse micellization. The inner surface of the membrane channels has negatively charged regions that allow cations to transit [29,30,31]. These channels have nanometer dimensions and the structure of these channels allows the separation of H^+^ and OH^−^ ions on both sides of the membrane [32,33]. In this regard, the Nafion membrane is actively used in low-temperature hydrogen fuel cells [34].

The creation of optical systems with controlled luminescence and the affordable practical realization of luminescent transducers are urgent tasks. For this reason, we investigated the process of embedding colloidal nanocrystals into the matrix of a Nafion proton exchange membrane. To obtain a new molecular complex we used already prepared membranes and colloidal CdSe/CdS/ZnS nanocrystals. The embedding process was carried out at room temperatures without disturbing the environmental conditions of the laboratory.

The aim of this work was not only to study the process of embedding colloidal CdSe/CdS/ZnS nanocrystals into the Nafion matrix, but also to determine the degree of the doping effect on the optical properties of the polymer membrane. The process of nanocrystal embedding into the membrane was controlled by luminescence, light absorption spectroscopy and laser emission spectroscopy techniques.

## 2. Materials and Methods

The colloidal nanocrystals (CdSe/CdS/ZnS) represent a core/shell/shell system structurally and have two type I heterojunctions. The nanocrystals used in this work were synthesized using a technique similar to that presented in [34,35,36]. The nanoparticles were stabilized with oleylamine (C_18_H_35_NH_2_). According to the results of electron microscopy performed on a transmission electron microscope JEOLJSM-7001F (JEOL, Tokyo, Japan) using ImageJ program (Version 1.52), the sizes of nanoparticles were estimated taking into account the passivation layer: 8.1 ± 0.2 nm for NC_1_, 8.3 ± 0.2 nm for NC_2_, 9.1 ± 0.2 nm for NC_3_. The standard deviation of the nanoparticle sizes was about 1.3 nm [34].

**Figure 1:**
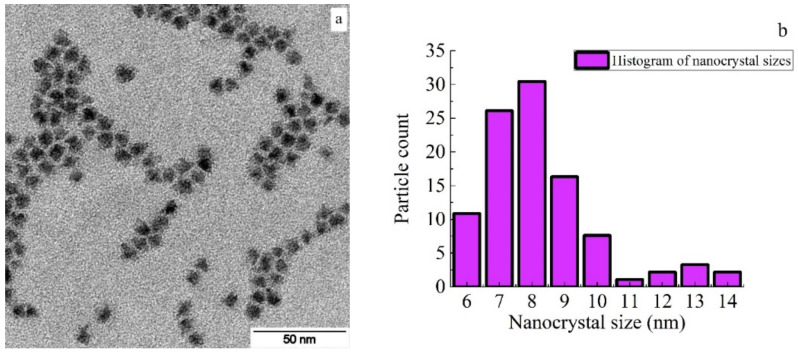
(**a**) Image of CdSe/CdS/ZnS nanocrystals obtained with transmission electron microscopy; (**b**) histogram for the size of CdSe/CdS/ZnS nanocrystals.

An image of CdSe/CdS/ZnS nanocrystals obtained using a transmission electron microscope JEOLJSM-7001F is shown in Figure 1a. The size distribution of the investigated CdSe/CdS/ZnS nanocrystals is shown in Figure 1b. According to calculations made using the ImageJ program for the measurements presented in Figure 1a, the average particle size was (8.8 ± 0.2) nm.

The embedding of colloidal nanocrystals (CdSe/CdS/ZnS) from solution into Nafion membrane was carried out by soaking the membrane in toluene solution (C_7_H_8_) with nanocrystals for 100 min and for 30 days. Nafion plate thickness was d = 175 µm. The volume of toluene solution with colloidal nanocrystals was 4 mL. Soaking of the membrane in the solution with nanocrystals was carried out for 100 min and for 30 days while the volume was thermostated at T = (22 ± 2) °C. The kinetics of the process of settlement of nanocrystals in the membrane was determined. For this purpose, the membrane was kept in solution and the panoramic absorption spectrum of the membrane was measured at 5 min intervals. Toluene was used as a means of suspending colloidal nanocrystals and allowing for further transport of them inside the polymer membrane. In doing so, toluene molecules activated the mobility of the sulfone groups of the membrane. The result of nanocrystal embedding was monitored by photoluminescence and with absorption spectra of the samples in the visible and UV wavelength ranges, as well as laser emission spectroscopy.

**Figure 2:**
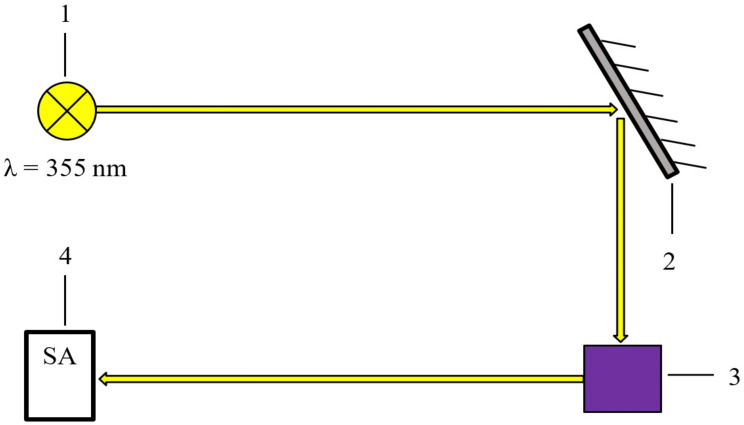
Block diagram of the experimental setup for luminescence registration: 1—neodymium laser on alumina garnet (QX500 SolarRS); 2—beam splitter; 3—sample; 4—secondary radiation receiver.

The luminescence spectra were registered using an experimental setup consisting of a laser radiation source consisting of a neodymium laser on alumina garnet (QX500 SolarRS, Solar Laser Systems, Minsk, Belarus) with a fundamental generation line at a wavelength of 1064 nm—1; a beam splitter—2; a sample—3; and a secondary radiation receiver—4 (Figure 2). A third harmonic of Nd^+3^: YAG laser with wavelength λ = 355 nm was used to excite luminescence in the studied samples. The laser operated in Q-switching mode and generated pulses with a duration of 10 ns and a repetition rate of 20 Hz. The mini-spectrometer (ASP-100) was a receiver of secondary radiation and allowed the registration of a useful signal in the range of 180–1100 nm with a spectral resolution of ≈0.3 nm (Figure 2).

Research of the optical membranes properties was carried out using spectrophotometer PB2201 (SOLAR, Solar Laser Systems, Minsk, Belarus) by recording the absorption spectra of the samples in the wavelength range of 190–900 nm. This spectrophotometer uses a two-beam scheme with base line recording and wavelength setting accuracy not more than 0.5 nm.

Laser emission spectroscopy was performed on the Laes Matrix Spectrometer. The LAES series instruments use a two-pulse solid-state laser as an excitation source (YAG: Nd^3+^ laser specifications: generated radiation wavelength: 1.064 μm; pulse repetition rate: 1–10 Hz; radiation pulse energy: at least 100 mJ/pulse) with Q-switching, which reduces the limiting concentrations of detection of elements and allows excitation of chemical elements with high ionization energy.

## 3. Results and Discussion

### 3.1. Luminescence Spectra of the Samples

**Figure 3:**
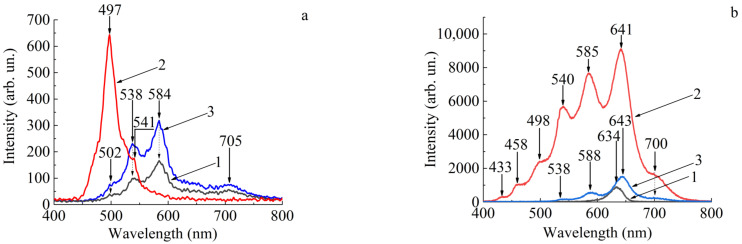
Luminescence spectra of the samples: (**a**) 1—Nafion membrane; 2—toluene; 3—Nafion membrane soaked in toluene; (**b**) 1—CdSe/CdS/ZnS nanocrystals; 2—Nafion membrane with embedded nanocrystals in the wet state of the membrane; 3—Nafion membrane with embedded nanocrystals in the dried membrane.

Figure 3a (curve 1) shows the luminescence spectrum of the original Nafion polymer membrane. The secondary emission maxima during radiation excitation of the untreated membrane are observed at wavelengths λ_Naf_ = 538, 584, 705 nm. The intensity maxima in the luminescence spectrum of toluene, which was used as a base for the preparation of the solution with colloidal nanocrystals, are observed at wavelengths of 497 and 541 nm (Figure 3a, curve 2).

The secondary emission spectrum of the membrane soaked in toluene is shown in Figure 3a, curve 3. The luminescence maxima of the Nafion membrane soaked in toluene for a long time are observed at wavelengths of 502, 538, 584, 705 nm. From the comparison of the spectral distribution of luminescence intensity maxima of the original sample (Figure 3a, curve 1) and the Nafion membrane soaked in toluene (Figure 3a, curve 3), it follows that the luminescence lines became more intense, but the positions of the spectral maxima remained practically unchanged.

The process of embedding nanocrystals into the Nafion membrane was carried out by placing the plate in a solution with colloidal nanocrystals suspended in toluene. Figure 3b, curve 1, shows the luminescence spectrum of a solution of semiconductor nanoparticles in toluene. The maximum of the secondary emission of quantum dots is detected at a wavelength of 634 nm (Figure 3b, curve 1). At the same time, the most intense spectral maximum of toluene (497 nm), which is shown in Figure 3a, curve 2, was not actively manifested in the system “toluene-nanocrystals”. The intensity of all the luminescence maxima of the toluene-nanocrystals system is greater than that of the initial toluene solution with nanocrystals.

The luminescence spectrum of the membrane, which was soaked for 30 days in the colloidal nanocrystal solution, is shown in Figure 3b (curve 2). The spectrum was measured immediately after the sample was extracted from the solution and the membrane was in a wet state when the secondary emission was recorded. Then, the membrane with embedded nanocrystals was dried in air and the luminescence spectrum was measured again in the same spectral range (Figure 3b, curve 3). The luminescence spectrum of the Nafion membrane with introduced nanocrystals after it had dried shows intensity maxima at wavelengths of 538, 588, 643 and 700 nm. Importantly, an additional maximum appears in the secondary emission spectrum of the dried membrane, which is observed at a wavelength of 643 nm. The luminescence maximum observed in the spectrum of CdSe/CdS/ZnS nanocrystals falls at a wavelength of 634 nm (Figure 3b, curve 1). In the dried membrane with embedded nanocrystals, this maximum is observed at a wavelength of 643 nm (Figure 3b, curve 3). Thus, the wavelength at which the luminescence maximum of the formed molecular structure obtained by embedding CdSe/CdS/ZnS nanocrystals into the Nafion membrane was observed shifted in wavelength by about 10 nm. The 643 nm photoluminescence line does not appear in the spectra of the solution components.

Seven pronounced maxima are observed in the luminescence spectrum of the Nafion membrane with embedded nanocrystals (Figure 3b, curve 2). An additional high-intensity spectral component of luminescence appears at a wavelength of 641 nm. In the dried membrane with embedded nanocrystals, the observed intensity maximum is shifted by 2 nm to the long-wave region of the spectrum (Figure 3b, curve 3). The luminescence maximum of nanocrystals suspended in toluene is observed at a wavelength of 634 nm (Figure 3b, curve 1). The intensity of the luminescence spectrum of the components of the “Nafion membrane–nanocrystals” system significantly increases compared to the secondary emission intensity of the initial solution components.

The number of lines in the luminescence spectrum produced by the membrane with nanocrystals in the wet state (Figure 3b, curve 2) is significantly larger than in the similar spectrum for the original membrane and the membrane in the dried state (Figure 3b, curve 3). Due to the prolonged presence of the membrane in toluene solution with colloidal nanocrystals, the luminescence intensity of such a system increases. It can be assumed that, as a result of toluene evaporation from the membrane surface, there is a deformation of the fluorocarbon membrane framework as perfluorinated vinyl ether chains containing clusters of sulfonate ions that have attached colloidal nanocrystals. As the sulfonate groups experiencing a Coulomb interaction in the nanocrystals move, the electrical potential of the system changes. As a result, additional phonon states are formed and the number of luminescence lines increases.

### 3.2. Optical Properties of the Samples

**Figure 4:**
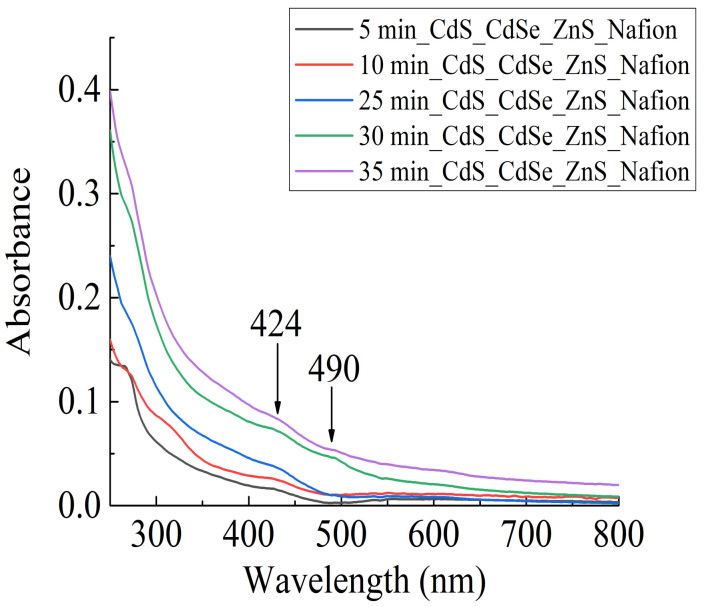
Absorption spectrum of the membrane in the process of nanocrystal embedding.

The kinetics of nanocrystal embedding into the membrane was investigated by measuring the membrane absorption spectra as a function of membrane exposure time in solution with colloidal nanocrystals at a fixed temperature of 22 °C. The absorption spectrum of the membrane after the introduction of colloidal nanocrystals into it changed significantly. Figure 4 shows panoramic absorption spectra of the membrane with embedded nanocrystals in the range of 250–800 nm and with time intervals of 5, 10, 25, 30 and 35 min from the beginning of nanocrystal embedding. The ordinate axis indicates the relative units of radiation absorption coefficient. At wavelengths of 424 nm and 490 nm, two new absorption maxima appeared. Investigations of radiation absorption by the membrane in the range of 250…800 nm indicate an increase in the absorption coefficient of the membrane with embedded nanocrystals in relation to the original membrane.

**Figure 5:**
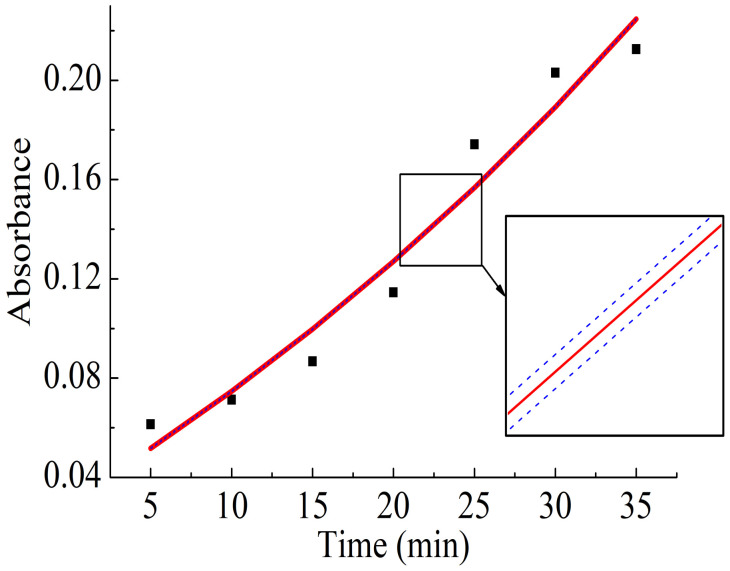
Time dependence of the membrane absorption coefficient at a wavelength of 300 nm.

Figure 5 shows the time dependence of the membrane absorption coefficient at a wavelength of 300 nm. The process of CdSe/CdS/ZnS nanocrystals embedding can be considered to be complete after 35 min, so the kinetic curve reaches its maximum. The relative radiation absorption intensity of the membrane with colloidal nanocrystals at 300 nm wavelength can be approximated as $0.23\cdot e^{\frac{t}{57.36}}-0.20$. The embedding rate of CdSe/CdS/ZnS nanocrystals into the Nafion polymer membrane was approximately 4·10^−3^ min^−1^. The inset in Figure 5 on the right shows the approximating curve taking into account the approximation error of 2.67·10^−4^ min^−1^.

**Figure 6:**
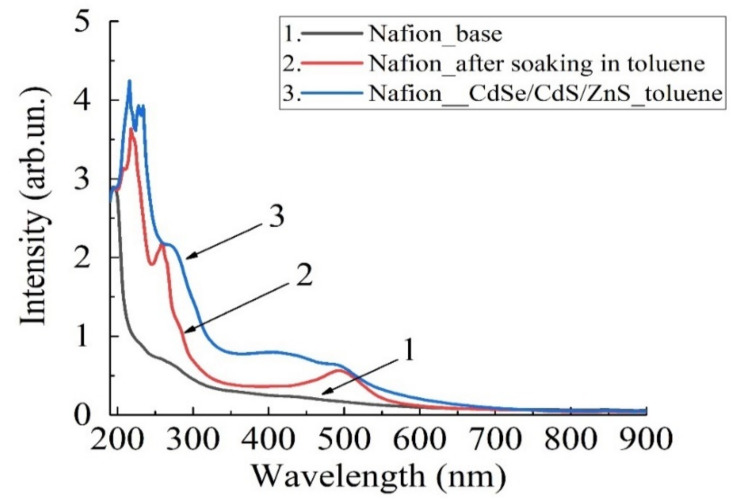
Absorption spectrum: 1—original Nafion membrane; 2—membrane after soaking in toluene; 3—membrane after soaking in toluene solution with nanocrystals.

Figure 6 shows the absorption spectra in the range (190…900) nm. The spectrum of the original Nafion membrane corresponds to curve 1 (Figure 6). The spectrum of the membrane soaked in toluene solution is shown by curve 2, and the spectrum of the membrane containing colloidal CdSe/CdS/ZnS nanocrystals corresponds to curve 3. It can be seen that the radiation absorption of the membrane increases. Using the data of the membrane absorption spectrum, we will be able to estimate the efficiency of nanocrystals introduction into the membrane and indirectly estimate the sizes of the formed clusters with nanocrystals.

The information on the distance between nanocrystals in the membrane is practically valuable. The calculation of such a distance requires non-empirical modeling of at least model fragments of the Nafion membrane, which was carried out for sodium, potassium, copper and sucrose salts in [37]. Based on the calculations in [37] and assuming that the average distance between sulfone groups is 15 Å, we have a cell area of 2.25·10^−18^ m^2^ in the planar approximation. For a Nafion plate with surface dimensions of 2 × 3 cm^2^ and a thickness of 175 μm, the total surface area would be 12.175·10^−4^ m^2^. In this case, the number of cells is approximately 5.4·10^14^—the number of potential centers on the membrane surface for embedding nanocrystals. The number of nanocrystals in the volume of 4 mL was 4.17·10^22^. Consequently, all sulfone groups located on the membrane surface have the potential to interact with nanocrystals, and the channels are filled with toluene. We can indirectly estimate the filling of membrane channels with toluene from the absorption spectra (Figure 6) of the membrane (curve 1) and the membrane soaked in toluene. The ratio of the areas under the absorption spectra in the range (190…900) nm for the membrane soaked in toluene solution (Figure 6, curve 2) and the original membrane (Figure 6, curve 1) was 1.82 times. The ratio of the areas under the absorption spectra in the range (190…900) nm for the membrane with nanocrystals in toluene (Figure 6, curve 3) and the original membrane (Figure 6, curve 1) was approximately 2.56. Thus, based on the above reasoning, it can be concluded that the entire surface of the membrane is occupied by nanocrystals.

**Figure 7:**
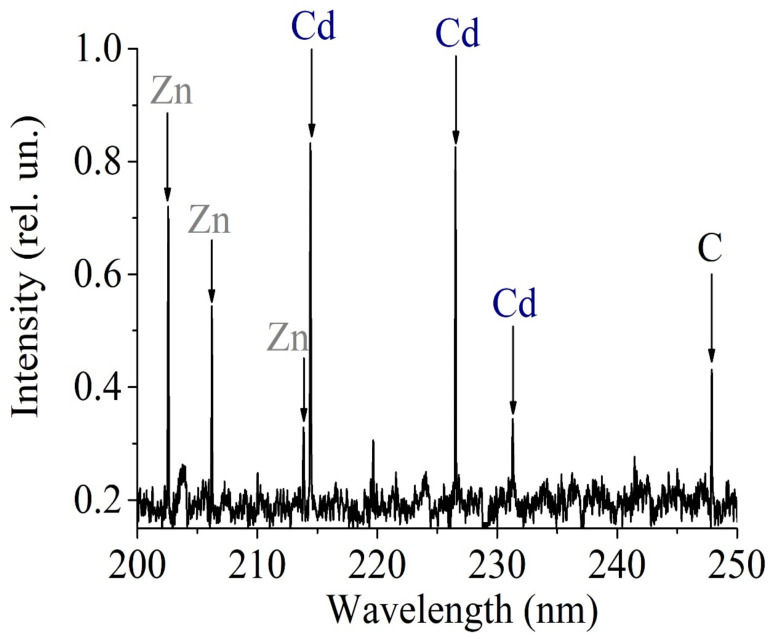
Emission spectrum of the membrane with CdSe/CdS/ZnS.

A direct and independent confirmation of the diffuse penetration of CdSe/CdS/ZnS nanocrystals into the membrane are the results of laser emission spectroscopy experiments obtained on the Laes Matrix Spectrometer. Atoms of the following chemical elements, Cd, Zn and C, were found to be present in the Nafion membrane (Figure 7).

Three series of spectral lines for Zn (202.5 nm, 206.2 nm, 213.8 nm), Cd (214.4 nm, 226.5 nm), as well as carbon, C, (248 nm) were detected (Figure 7). The method of obtaining the registered radiation in the process of laser emission spectroscopy and processing of the obtained lines do not allow us to determine the amounts of fluorine (F) and sulfur (S) present in the polymer membrane fibers.

**Figure 8:**
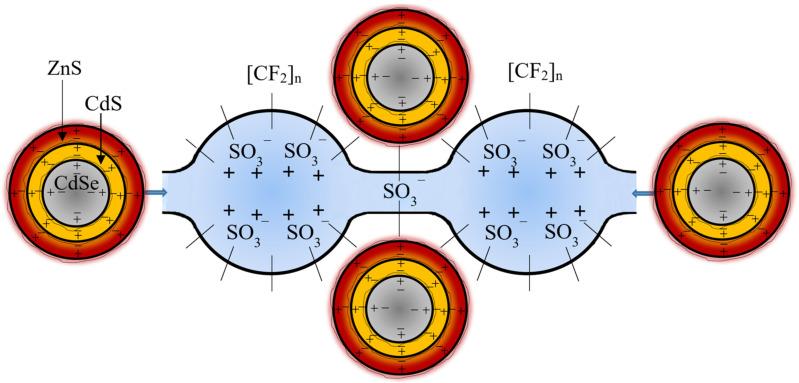
Schematic diagram of Nafion membrane links and colloidal CdSe/CdS/ZnS nanocrystals.

In accordance with [34], the size of CdSe/CdS/ZnS nanocrystals is approximately 8.1 nm. Research on the internal structure of the Nafion membrane shows that the polymer base (hydrophobic phase) consists of fluorocarbon and ether chains. Functional sulfo-groups are grouped inside spherical cavities with a diameter of about 40 Å. Figure 8 shows the scheme of arrangement and possible interaction of membrane links and colloidal CdSe/CdS/ZnS nanocrystals. The hydrophilic phase of the membrane is represented by a system of cavities connected by narrow channels, usually containing hydrated cations [23], the structure of which can accommodate CdSe/CdS/ZnS nanocrystals (Figure 8). The sign «−» denotes a negative charge, and the sign «+» denotes a positive charge (Figure 8). Along with diffusion processes, Coulomb interaction takes place in the system under consideration. The sulfone groups of the Nafion membrane, being in the state of dissociation and having a negative charge, cause the movement of colloidal nanocrystals due to Coulomb interaction with them.

The change in the position of the luminescence maximum of the secondary emission of the nanocrystals introduced into the membrane, which is 9 nm (Figure 3b, dependences 1, 3), means a change in the height of the potential relative to the surroundings, as well as a change in the effective size of the nanocrystals in the surroundings of the massive membrane molecular complex. According to the assessment, the diameter of CdSe and CdS nanocrystals has increased. This leads to the fact that the exciton localization region in nanoparticles increases [35].

**Figure 9:**
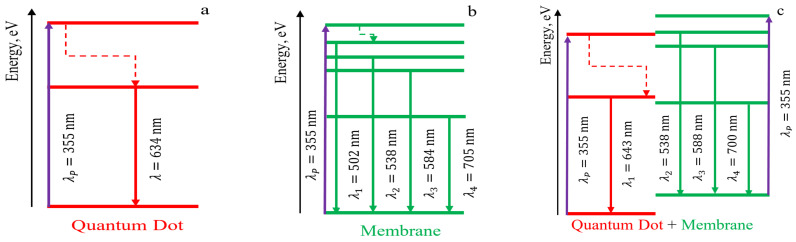
Structure of energy levels of the investigated systems: (**a**) CdSe/CdS/ZnS nanocrystals; (**b**) Nafion membrane; (**c**) dried Nafion membrane with introduced CdSe/CdS/ZnS nanocrystals.

**Figure 10:**
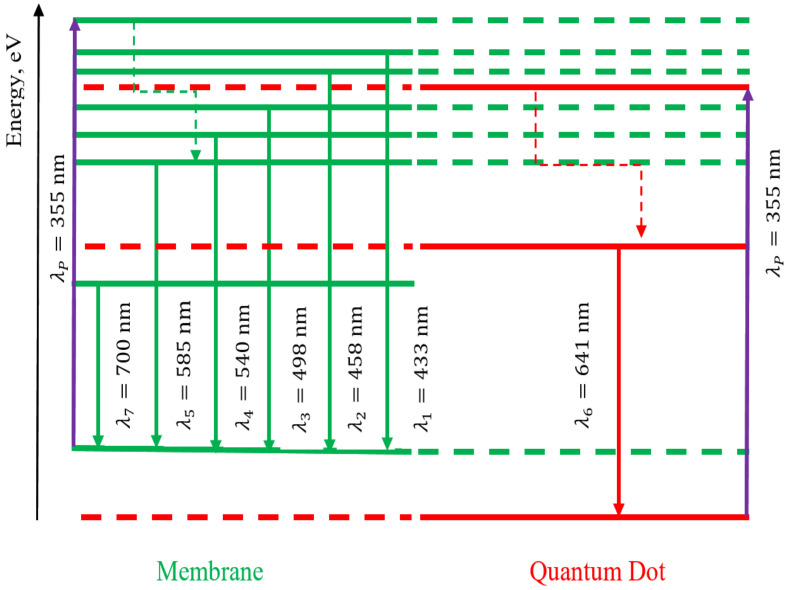
Schematic of energy levels of the wet Nafion membrane with introduced CdSe/CdS/ZnS nanocrystals: solid arrows indicate radiative transitions, and dashed mean non-radiative transitions.

Thus, the Nafion polymer membrane acts as a matrix, which together with nanocrystals, forms an electronic structure different from the electronic structure of colloidal nanocrystals. To explain this fact, we propose schematics of possible electronic transitions, which can be seen in Figure 9 and Figure 10.

Figure 9 schematically shows possible electronic transitions observed during luminescence: (a) for CdSe/CdS/ZnS nanocrystals; (b) for the initial Nafion membrane; (c) for the Nafion membrane with introduced CdSe/CdS/ZnS nanocrystals after membrane drying. Electron transitions without radiation (radiationless transitions) are marked in Figure 9 and Figure 10 by dashed lines with arrows.

Figure 10 shows possible radiative transitions due to excited electronic levels for the luminescence spectrum of wet Nafion membrane with introduced nanocrystals and non-radiative transitions (dashed lines with arrows). It can be noted that due to the change in the mass of the formed molecular complex and the change in the size of nanocrystals, there was a shift in the luminescence lines compared to Figure 3a. The luminescence spectrum of the wet membrane shows additional intensity maxima at wavelengths of 433 nm and 458 nm, which were not observed before. The appearance of the luminescence band in Figure 3b (dependence 2) in the wavelength range 490–590 nm is similar to the intensity distribution of the original and soaked Nafion (Figure 3a, dependences 1,3). According to the appearance of the observed dependence in Figure 3b (dependence 2), it is natural to assume that the new spectral components characterized by a monotonic decrease in intensity relative to the longer wavelength peak in the region of 433 nm and 458 nm are related to radiative transitions from excited electronic levels of the membrane.

The sharp increase in the luminescence intensity of the wet membrane with introduced nanocrystals relative to the dried sample and the appearance of new spectral components can be related to the achievement of a higher power density of excitation radiation in a more optically dense medium due to the content of toluene in the membrane, the refractive index of which is 1.49.

We have analyzed the features of the luminescence spectrum of the membrane populated with colloidal nanocrystals from the viewpoint of the electronic structure of the system. Due to a larger number of excitation photons, the occupancy of shorter-wavelength energy levels increases, which is manifested as low-intensity satellites at 433 nm and 458 nm. The secondary emission intensity of the membrane with introduced nanocrystals is greater than that of the original membrane and the nanocrystals themselves (Figure 3). The luminescence line width for the colloidal solution of nanocrystals in toluene is twice as large compared to the luminescence line width of the “Nafion membrane–nanocrystals” system. The increase in the intensity of secondary emission can also be observed as a result of resonance excitation of the substance’s own exciton level [38] or the impurity center of the complex under investigation [39]. In this case, the luminescence spectrum takes the form of a broad band consisting of a large number of equidistant intensity maxima, which correspond to the manifestation of optical phonons of the substance, which is important for the practical use of the complex “Nafion–colloidal CdSe/CdS/ZnS nanocrystals” in optical systems.

To estimate the penetration depth of colloidal CdSe/CdS/ZnS nanocrystals into the Nafion membrane, we used the diffusion theory [40,41]:

(1) $$\frac{1}{D_{v}}\frac{\partial C_{v}}{\partial t}=∆C_{v}$$

The period of the nanocrystals’ interaction with the membrane exceeds the characteristic time $\tau =\frac{R^{2}}{D_{v}}$ [42], which is of the order of $72.25\cdot 10^{-6}\;s$. It is taken into account here that the diameter of quantum dots in Nafion is 16.20 nm and the diffusion coefficient $D_{v}\approx 10^{-12}\frac{m^{2}}{s}$. The process of keeping the membrane in solution with colloidal nanocrystals was not time-limited. Therefore, if the condition t ≫ τ is fulfilled, we can assume that $\frac{1}{D_{v}}\frac{\partial C_{v}}{\partial t}=0$. For the one-dimensional case with a plate of thickness d, from the equation $∆C_{v}=0$, the concentration distribution of colloidal nanocrystals will be defined as:

(2) $$C\left(x\right)=C_{max}\left(1-\frac{2}{d}x\right)$$

Unfortunately, the depth of the nanocrystals’ penetration into the membrane could not be experimentally established. However, luminescence and laser emission spectroscopy experiments confirm a rather active interaction between the sulfone groups of the membrane and nanocrystals.

## 4. Conclusions

The results of luminescence, absorption and emission spectroscopy experiments indicate the creation of a molecular complex based on the proton exchange membrane Nafion and colloidal nanocrystals of CdSe/CdS/ZnS type. In this case, the Nafion membrane acts as a matrix, which together with nanocrystals forms an electronic structure different from the electronic structure of colloidal nanocrystals.

The characteristics of the luminescence spectrum of the membrane with nanocrystals differ significantly from the spectrum of the original membrane, solvent and embedded CdSe/CdS/ZnS nanocrystals.

The absorption of optical radiation of the membrane with embedded nanocrystals in the range (190…900) nm increases (Figure 4 and Figure 6), the formation of new spectral lines of radiation absorption is observed. Practically all sulfo-groups located near the membrane surface form bonds with colloidal nanocrystals.

In fact, the polymer proton exchange membrane with embedded nanocrystals is a luminescent transducer that is able to convert UV radiation into visible radiation. Such a transducer can be used to create a luminescent balneological dressing in medicine or can be used in plant growing and storage systems as a covering material. An increase in the intensity of the luminescence spectrum and the appearance of additional luminescence lines in the “Nafion–colloidal nanocrystals CdSe/CdS/ZnS” system can be achieved by using the wet state of the membrane.

In conclusion, we note that the proton transfer membrane with luminescence centers in the form of CdSe/CdS/ZnS nanocrystals is an element of an optical system that can be used to increase the conversion efficiency of light energy into electrical energy by photocathodes and photodiodes, and to change the resistance of photoresistors. The introduction of colloidal CdSe/CdS/ZnS nanocrystals into the Nafion membrane will allow us to create a new generation of conversion and correction light filters. When solar cells and conversion light filters that convert UV radiation into visible and diffuse IR radiation are used together, it is possible to reduce the heating of solar cells and increase the efficiency of solar cells.

## Data Availability

The data presented in this study are available on request from the corresponding author.
